# Redefining Diagnostic Cut‐Offs for the Indirect Water Deprivation Test

**DOI:** 10.1111/cen.15172

**Published:** 2024-12-05

**Authors:** Yash Akkara, Kavita Narula, Katharine Lazarus, Deborah Papadopoulou, Sirazum Choudhury, Niamh Martin, Karim Meeran

**Affiliations:** ^1^ Division of Diabetes, Endocrinology and Metabolism, Department of Metabolism Digestion and Reproduction Imperial College London London UK; ^2^ Department of Endocrinology Imperial College Healthcare NHS Trust London UK; ^3^ Department of Clinical Biochemistry North West London Pathology London UK

**Keywords:** dynamic function testing, osmolality, sodium, vasopressin deficiency, water deprivation test

## Abstract

**Objective:**

An incorrect diagnosis of arginine vasopressin deficiency and resistance (AVP‐D and AVP‐R) results in the potentially dangerous use of desmopressin in healthy individuals. The water deprivation test is a central diagnostic test in patients with polydipsia polyuria syndrome (PPS). This study aims to determine the effectiveness of the current interpretation of reference ranges.

**Methods:**

This is a retrospective analysis of 135 patients who underwent a water deprivation test between August 2014 and August 2023. All patient diagnoses were reviewed, and variability and receiver operating characteristic (ROC) curves were determined for serum osmolality, serum sodium and urine osmolality.

**Results:**

Serum sodium demonstrated reduced variability compared with serum osmolality (0.72% vs. 1.16%, respectively, 37.5% reduction; *p* < 0.001). The standard serum osmolality cut‐off value of ≥ 300 mOsm/kg in diagnosing AVP‐D, AVP‐R, and primary polydipsia (PP) achieved a sensitivity of 76.19% and specificity of 76.92%. A serum sodium cut‐off value of ≥ 148 mmol/L demonstrated 100% specificity in excluding PP. This cut‐off was used alongside urine osmolality cut‐off values of > 630 mOsm/kg (for PP) and < 383 mOsm/kg (for AVP‐D/AVP‐R). Review of post‐desmopressin urine osmolality and clinical monitoring was performed in equivocal diagnostic cases (*n* = 6), achieving 100% sensitivity and 100% specificity within the study sample.

**Conclusions:**

This study demonstrates that a serum sodium cut‐off of ≥ 148 mmol/L in tandem with urine osmolality yields the best diagnostic accuracy to differentiate between AVP‐D, AVP‐R, and PP. Serum sodium may be more reliable than serum osmolality in the investigation of patients with PPS, demonstrating lower biological and analytical variability.

## 1 Introduction

The indirect water deprivation test is the gold standard to differentiate between arginine vasopressin deficiency (AVP‐D), arginine vasopressin resistance (AVP‐R), and psychogenic polydipsia (PP) in patients with polydipsia‐polyuria syndrome [1, 2, 3]. During this investigation, the patient is deprived of fluids for 8–10 h, with regular measurements of urine volume, and urine and serum osmolality.

In healthy individuals, urine osmolality is expected to rise to (and above) 750 mOsm/kg during the fluid deprivation phase, with little to no increase following desmopressin administration. In patients with complete AVP‐D, urine osmolality is expected to stay below 300 mOsm/kg for the fluid deprivation phase but is expected to rise by greater than 50% following desmopressin. For patients with AVP‐R, urine osmolality also remains below 300 mOsm/kg for the fluid deprivation phase but does not rise by more than 50% following desmopressin, owing to renal resistance [4].

Measurement of serum osmolality in the first phase of the indirect water deprivation test aims to identify the ability to maintain osmotic balance within physiological limits. Serum osmolality levels of less than 280 mOsm/kg are indicative of PP whereas an osmolality of greater than 300 mOsm/kg is diagnostic of AVP‐D or AVP‐R [5, 6, 7, 8]. The test is stopped if the patient loses more than 3% of baseline body weight as excessive dehydration can be dangerous, and this amount of weight loss is indicative of a positive test.

Osmolality is measured by freezing point depression, but can also be estimated from measured sodium, potassium, urea, and glucose. The latter three change very little, so the major influence on serum osmolality is serum sodium. Hence, we hypothesised that serum sodium would be a more reproducible measurement than serum osmolality. This study therefore aimed to investigate the diagnostic accuracy of the ≥ 300 mOsm/kg serum osmolality cut‐off in comparison to serum sodium, particularly in differentiating AVP‐D and AVP‐R from patients with physiologically normal AVP and/or renal function.

## 2 Methods and Materials

This was a retrospective study of all water deprivation tests performed in adults at Imperial College Healthcare NHS Trust (ICHNT) London over a 9‐year period. Clinical and biochemical data from the water deprivation test protocol, including periodic measurements of urine volume, urine osmolality, serum osmolality, serum sodium and body weight were obtained from patient electronic records. Consistent with departmental protocol, patients with an inconclusive result underwent a prolonged water deprivation test.

The procedures for the standard and the prolonged water deprivation test were carried out according to published protocols [6, 9, 10]. In brief, during the first phase of water deprivation, four serum (P_1_ to P_4_) and urine (U_1_ to U_4_) samples were collected at regular intervals. Following this, intramuscular desmopressin was administered, after which four additional urine samples (U_5_ to U_8_) were collected. Serum samples were used to measure serum osmolality and sodium, and urine samples were used to measure urine osmolality.

During the prolonged test, monitoring was carried out as described above for the standard test [6]. Urine samples were collected and measured hourly, with serum samples collected and measured every 2 h. Water deprivation was continued until three consecutive urine osmolality measurements increased by less than 30 mOsm/kg. Intramuscular desmopressin was then given, with four consecutive urine measurements collected at hourly intervals. For safety, both the standard and prolonged tests were stopped in the event of greater than 3% weight loss, significant changes in blood pressure, or other systemic signs.

The final diagnosis was confirmed following a comprehensive clinical review by a panel of endocrinologists based on the WDT and clinical history of patients. All patients were followed‐up for at least 4 months. Patients were classified as positive (i.e., a diagnosis of AVP‐D and AVP‐R), and negative in those without disorders of vasopressin (normal and PP). Partial cases generally constituted patients whose urine osmolality was between 300 and 750 mOsm/kg with plasma osmolalities less than 300 mOsm/kg [10], alongside those who fell within this subtype based on clinical monitoring of symptoms and treatment needs. In cases where results were equivocal, repeat testing was performed. In further equivocal cases where repeat tests remained ambiguous, clinical review with desmopressin replacement was conducted. Patients who remained well without requiring desmopressin were considered healthy. Patients who required desmopressin replacement, were classified as having AVP‐D or AVP‐R (including partial cases). This study was completed as a registered service evaluation within ICHNT (ref: END_027).

### 2.1 Assay Methodology

Serum sodium concentrations were determined using either Abbott Architect or Alinity analysers. The intra‐assay and inter‐assay coefficients of variation for the Architect platform are < 1.4% and < 1.4%, respectively. The intra‐assay coefficient of variation (CV) for the Alinity platform is < 0.5%. We initially used the Abbott Architect platform until a phased transition across all sites to the Alinity platform occurred in 2018 to 2019. During this transition, the Alinity analysers were validated against the previous Architect analysers in compliance with ISO15189. The results generated on the Alinity analysers are directly comparable to the previous Architect analysers.

Serum and urine osmolality were measured using the freezing‐point osmometry method on an Advanced Instruments Model 3320 Micro‐Osmometer, with imprecision values of ±2 mOsm/kg up to concentrations of 400 mOsm/kg in serum and urine. The intra‐assay coefficient of variance (CV) was determined locally to be < 0.8% and < 0.9% for serum and urine respectively.

### 2.2 Statistical Analysis

Patients were grouped into distinct categories, based on their water deprivation test results and final diagnosis. These comprised: AVP deficiency, AVP resistance and healthy individuals.

Data were analysed using GraphPad Prism Version 10.1.1 for Mac (GraphPad, San Diego, CA).

The mean and standard deviation for all four plasma and urine osmolality, and serum sodium measurements were computed to derive the CV for each indicator across each patient. The CVs for each patients’ sodium were then compared to the CVs for their osmolality to identify which indicator was more variable.

Area under the curve (AUC) was calculated for the receiver operating characteristic (ROC) curve as a means of identifying improved thresholds for urine and serum osmolality, and serum sodium.

Data was assessed for normality. Parametric variables are presented as mean (±standard deviation). Nonparametric data sets are presented as median (interquartile range). *p* < 0.05 was considered statistically significant.

## 3 Results

### 3.1 Study Sample and Demographics

**Figure 1:**
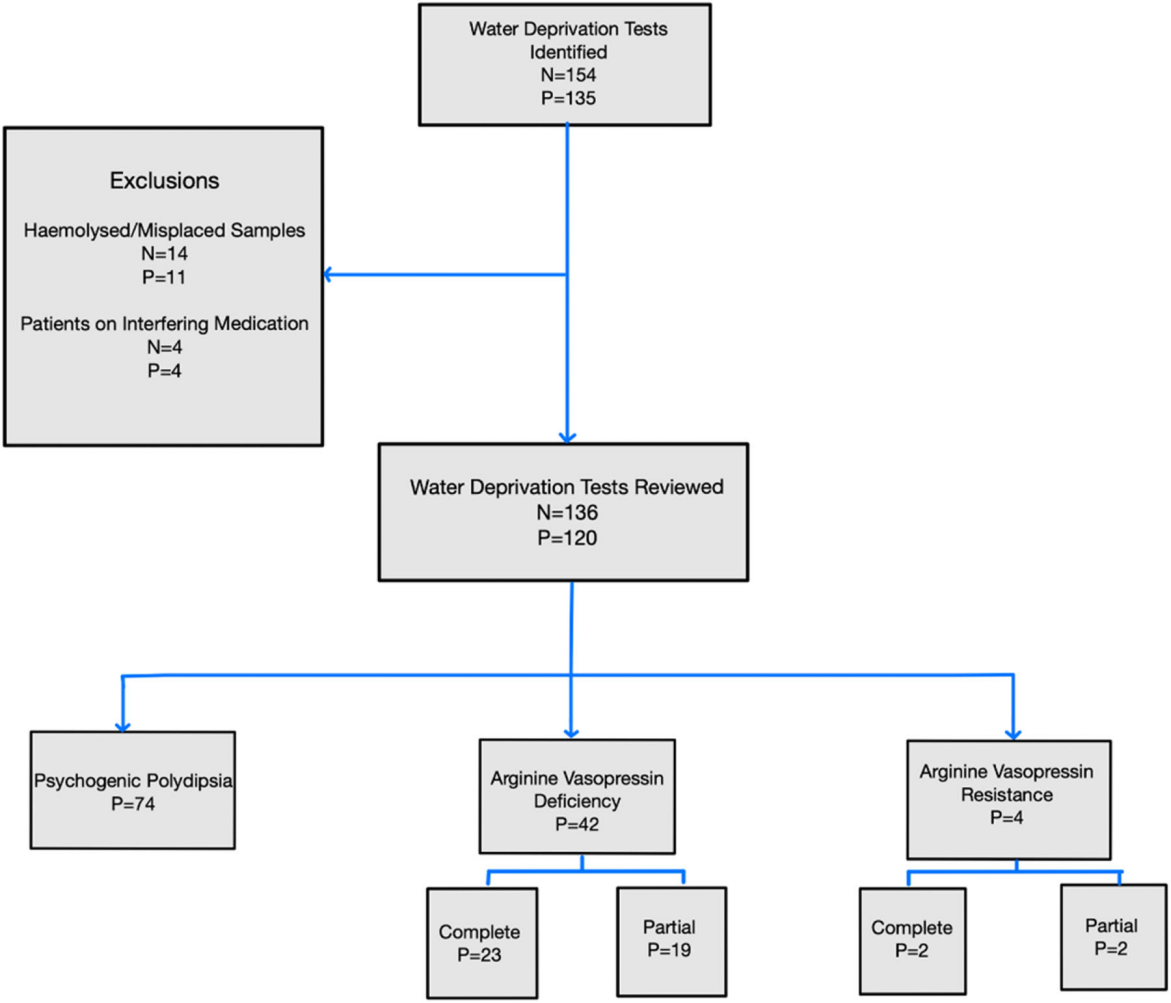
STROBE diagram of the study design. N = number of tests, P = number of patients.

In total, 154 tests in 135 individuals were completed over the 9‐year review period. Twenty‐nine patients had an inconclusive water deprivation test and were referred for a prolonged water deprivation test. Eighteen tests were excluded from the analysis owing to haemolysed or misplaced samples, or the presence of interfering medication. Of those remaining, a total of 136 tests from 120 patients were included in the study (Figure 1).

### 3.2 Variance of Serum Osmolality and Serum Sodium

The mean and standard deviation for serum osmolality and serum sodium were calculated using the four serum measurements (P_1_, P_2_, P_3_, P_4_). The CV was then obtained by dividing the standard deviation of each patient's measurements by the respective mean and expressed as a percentage value.

**Figure 2:**
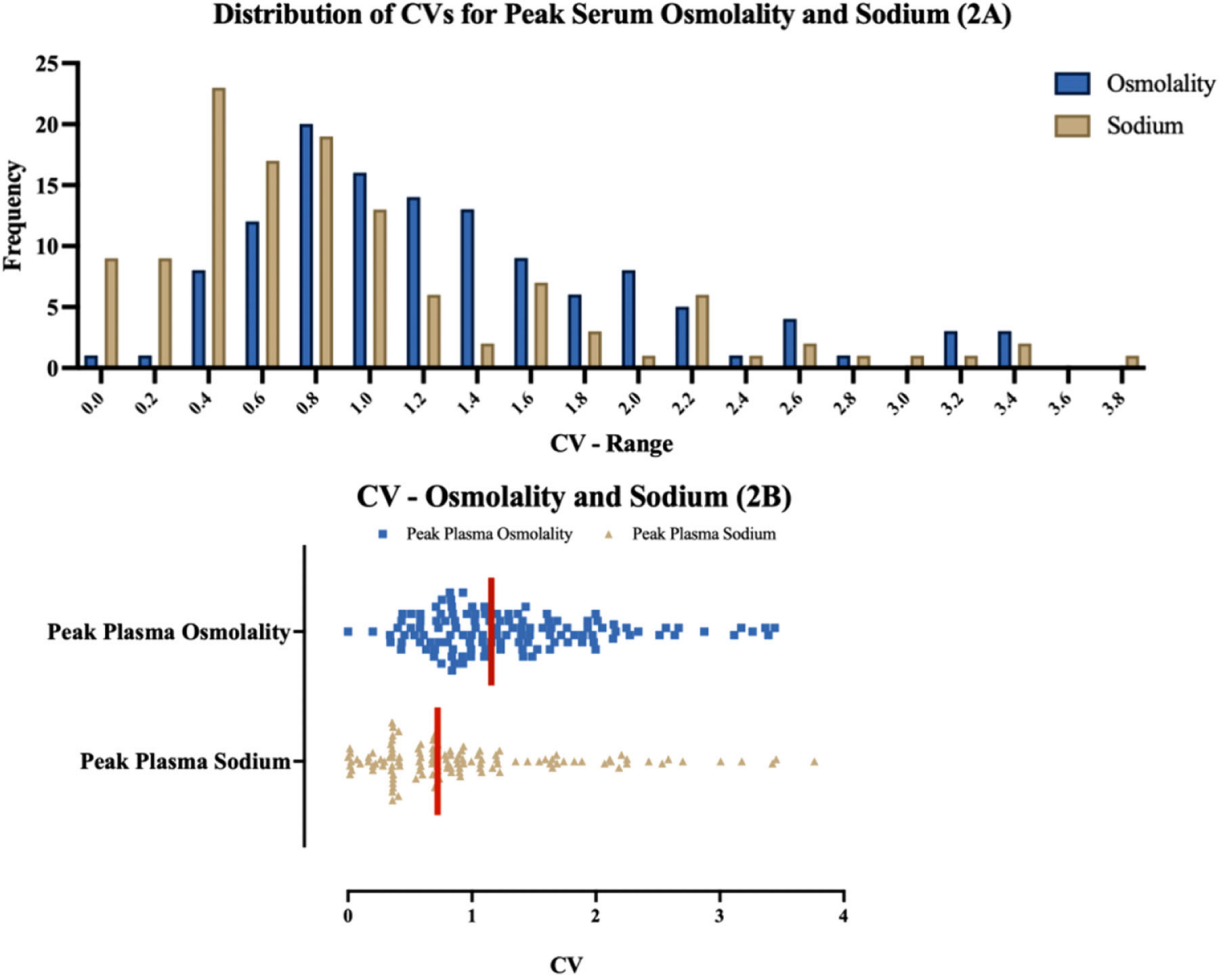
(A) Frequency distribution of CVs for serum osmolality and sodium. (B) Scatterplot of serum osmolality and sodium CVs. The vertical red line in 2B denotes the median.

Figure 2 shows the respective distribution of the CVs for all tests between serum osmolality and sodium. The median percentage CV for peak serum osmolality was 1.16% (0.888), and 0.72% (0.843) for peak serum sodium. This, along with the frequency distributions, demonstrates peak serum sodium is significantly less variable by 37.5% (*p* < 0.001) compared to peak serum osmolality.

### 3.3 ROC Analysis of Peak Serum Sodium, Serum Osmolality and Urine Osmolality During the Indirect Water Deprivation Test

**Figure 3:**
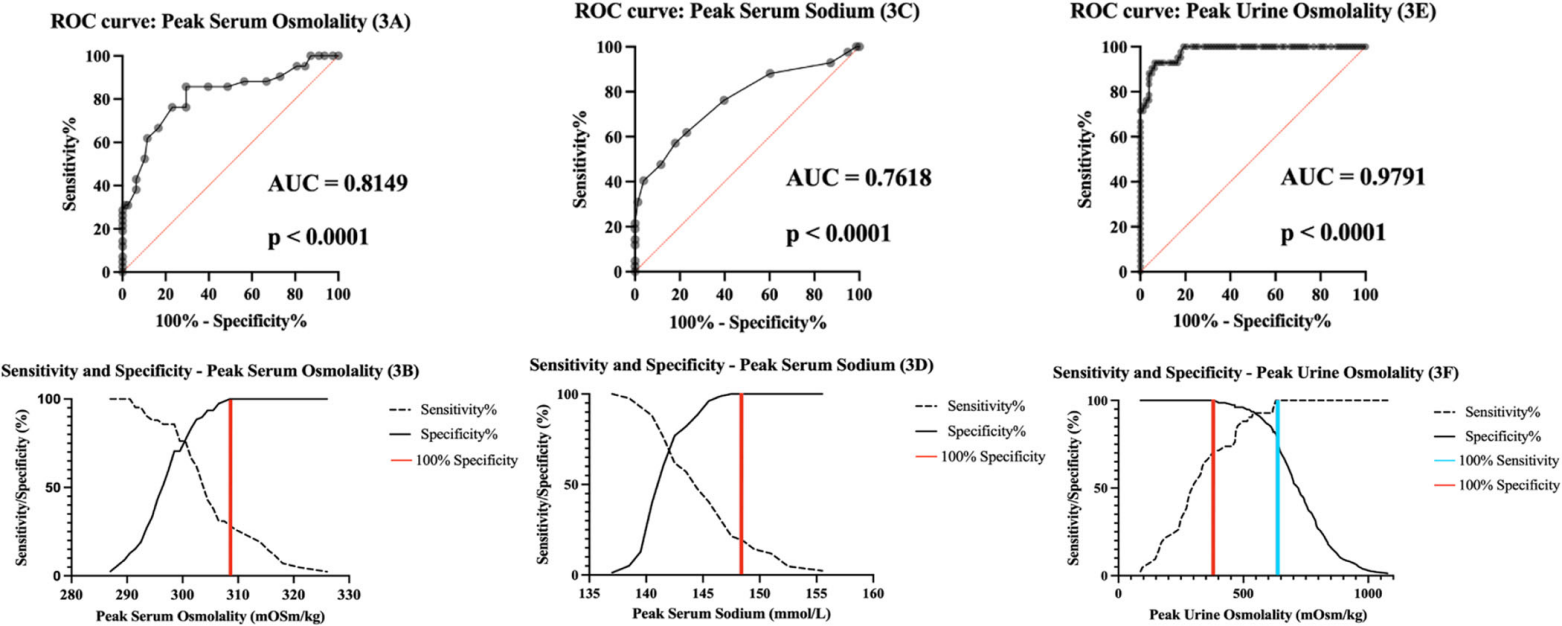
Receiver operating characteristic (ROC) and sensitivity/specificity curves for Phase 1 of the indirect water deprivation test: (A and B) ROC and sensitivity/specificity curves for peak serum osmolality. (C and D) ROC and sensitivity/specificity curves for peak serum sodium. (E and F) ROC and sensitivity/specificity curves for peak urine osmolality. Area under the curve (AUC) values have been labelled on the graphs.

The sensitivity and specificity of the current cut‐off values were calculated for peak serum osmolality, peak serum sodium, and peak urine osmolality and plotted on a ROC curve. This produced AUC values of 0.815 (peak serum osmolality, *p* < 0.0001), 0.762 (peak serum sodium, *p* < 0.0001) and 0.979 (peak urine osmolality *p* < 0.0001), respectively (Figure 3).

ROC curves were also generated for changes in urine osmolality changes across the entire sample during the second phase of the test and for patients with a positive diagnosis of AVP‐D or AVP‐R. Within the subgroup of patients with a positive diagnosis, patients with AVP‐R were considered as having a negative test, and those with AVP‐D as having a positive test result.

In the ROC analysis, based on the clinical relevance of differences in treatment, individuals with the diagnosis of AVP‐D and AVP‐R (including partial diagnoses) were grouped as positives, whereas patients with PP or normal AVP function were grouped as negatives. Use of the standard serum osmolality cut‐off of ≥ 300 mOsm/kg, demonstrated a sensitivity of 76.19% (patients who were negative and were identified correctly) and specificity of 76.92% (patients who were positives and were identified correctly). Applying this to the sample patient population results in 19.2% of patients being classified as false positives.

### 3.4 Updated Cut‐Off Values

Increasing the serum osmolality cut‐off to > 307.5 mOsm/kg demonstrates 100% specificity with 28.57% sensitivity (Table 1). This reduces the number of false positives from 23 (with the osmolality cut‐off) to zero and increases the number of true negative individuals (healthy patients being correctly identified) by 41.8% (55 individuals using a cut‐off of > 300 mOsm/kg compared to 78 using a cut‐off value of > 307.5 mOsm/kg).

**Table 1:** Sensitivity and specificity cut‐offs for serum osmolality, sodium, and urine osmolality.

ROC—Phase 1 findings			
	Peak serum osmolality (mOsm/kg)	Peak serum sodium (mmol/L)	Peak urine osmolality (mOsm/kg)
100% Sensitivity			
Threshold/cut‐off	< 290.5	< 137.0	> 630.0
Specificity%	12.82%	1.282%	80.77%
100% Specificity			
Threshold/cut‐off	> 307.5	> 147.5	< 383.0
Sensitivity%	28.57%	21.43%	71.43%

The cut‐offs for peak serum sodium are < 137.0 mmol/L (100% sensitivity) and > 147.5 mmol/L (100% specificity). The cut‐offs for peak urine osmolality are > 630.0 mOsm/kg (100% sensitivity) and < 383.0 mOsm/kg (100% specificity).

The ROC analysis of the percent‐change in urine osmolality following desmopressin also reveals distinct cut‐offs. In both patient groups (positive and negative patients), a reduction of 2% or more in urine osmolality demonstrates 100% sensitivity in differentiating AVP‐D and AVP‐R from PP; whilst an increase of greater than 53.4% demonstrates 100% specificity when considering AVP‐D and AVP‐R as the positives (Table 2).

**Table 2:** Specificity and sensitivity cut‐off values for the percent‐changes in urine osmolality following desmopressin administration.

ROC—Phase 2 Findings		
	Urine osmolality changes—Complete sample (*N* = 120)	Urine osmolality changes—Positives (AVP‐D and AVP‐R), with AVP‐D defined as a positive result (*N* = 46)
100% Sensitivity		
Threshold/cut‐off	< −2.00%	< 23.37%
Specificity%	9.46%	0.00%
100% Specificity		
Threshold/cut‐off	> 53.40%	> 54.00%
Sensitivity%	59.09%	65.00%

*Note:* The middle column (complete sample) demonstrates the values for the entire study cohort and the column on the far right shows those patients within the positive patient cohort (with a diagnosis of AVP‐D and AVP‐R).

### 3.5 Equivocal Cases

Of the 120 patients, six (5%) did not meet both the serum sodium cut‐off value of ≥ 148 mmol/L and < 137 mmol/L, and a urine osmolality cut‐off value > 630 mOsm/kg and < 383 mOsm/kg. All equivocal cases underwent repeat testing, which was conclusive in four of the six cases. The remaining two patients were clinically reviewed based on their symptomatic benefit with desmopressin to make a diagnosis. Across these six patients, four patients were identified as having PP, and two patients were identified as having partial AVP‐D.

## 4 Discussion

This study demonstrates that measured serum sodium has a significantly lower variability between measurements compared to serum osmolality during water deprivation. As the criteria for serum osmolality is based on its peak which can occur across the four measurements, random variance in one measured value can lead to serum osmolality exceeding the defined diagnostic cut‐off, resulting in a positive diagnosis. Cut‐off values inherently exist to terminate tests and identify true positives early. Variability in measurements can therefore undermine the effectiveness of the cut‐off by reaching the threshold due to random variation. This can be minimised by using a variable with inherently lower levels of variation in measurement, such as serum sodium. While there is an inherent level of variation stemming from the increase between P_1_ and P_4_ that is expected to result in similar CVs for both serum sodium and osmolality, the presence of significantly higher variability in serum osmolality suggests the presence of other factors that add random and unwanted variance to its measurement. One possible example for this could be that osmolality is measured using freezing point osmometry, which has a higher intra‐assay CV (< 1.4%) compared to the measurement of serum sodium (< 0.5%, which could result in increased variation compared to serum sodium over the course of the test.

The currently used serum osmolality cut‐off of > 300 mOsm/kg results in a high rate of false positive results (i.e., patients with PP wrongly classified as having AVP‐D, AVP‐R, or partial cases) in this study. An incorrect diagnosis leads to unnecessary and potentially dangerous use of desmopressin in healthy individuals, with the potential to induce adverse effects, including severe hyponatraemia.

Use of a serum sodium cut‐off of ≥ 148 mmol/L improves the specificity of the indirect water deprivation test to 100%. This reduces the number of false positives in this study from 23 to 0 and increases the number of true negative diagnoses by 41.8%. This is in keeping with the current literature; others have suggested serum sodium cut‐offs ranging from ≥ 146 mmol/L to > 150 mmol/L being diagnostic of AVP‐D or AVP‐R [4, 8].

Whilst using a serum osmolality cut‐off value of ≥ 308 mOsm/kg also demonstrates 100% specificity, the reduced inherent variability of measuring serum sodium means there is a lower likelihood of exceeding the cut‐off value due to random variation. This study demonstrates that a serum sodium cut‐off of ≥ 148 mmol/L can be used as a reliable marker of AVP‐D and AVP‐R with a specificity of 100%. Although serum osmolality has a slightly higher diagnostic accuracy independently (Table 1), its use is limited by increased variation, combined with reduced availability and higher turn‐around times [11].

Using the derived urine osmolality cut‐offs of < 383 mOsm/kg (to diagnose complete AVP‐D and AVP‐R) and > 630 mOsm/kg (to diagnose PP) in tandem with a serum sodium cut‐off of ≥ 148 mmol/L reduced the number of false negative diagnoses to zero. Within this study cohort, use of these cut‐off values allows a diagnosis of AVP‐D, AVP‐R and PP with 100% specificity and sensitivity. These results also suggest that a WDT may be avoided if a patient has a serum sodium value of greater than 148 mmol/L or a urine osmolality of greater than 630 mOsm/kg if water deprivation has coincidentally occurred.

**Figure 4:**
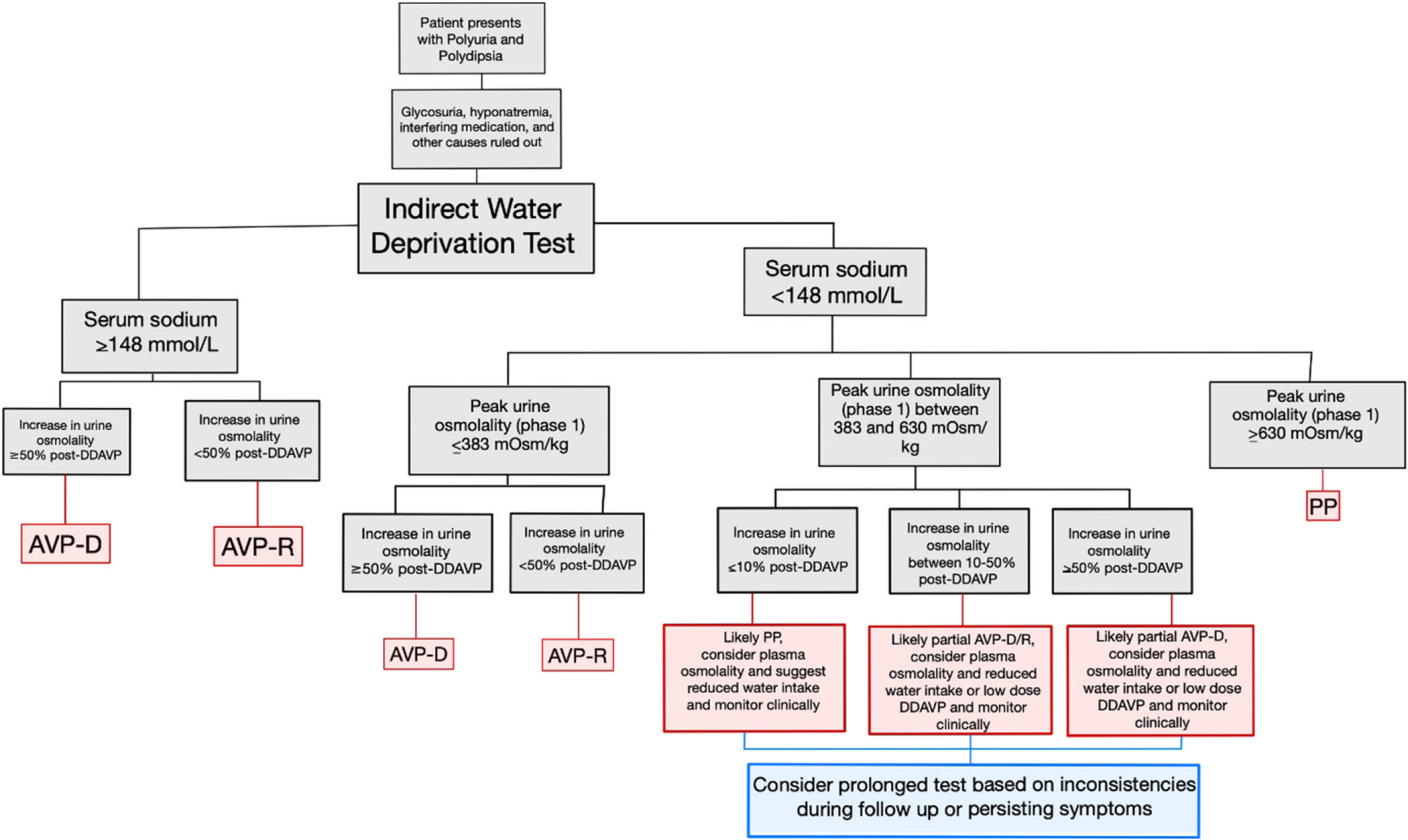
Summarised diagnostic approach in a patient presenting with polydipsia and polyuria.

Similarly, when evaluating the change in urine osmolality following desmopressin for the entire sample, 100% specificity is achieved with using a greater than 53.40% rise in urine osmolality. Within those patients with a positive diagnosis of AVP‐D and AVP‐R (including partial cases of both), 100% specificity for AVP‐D patients is achieved with at least a 54% increase in urine osmolality, consistent with an expected value of > 50% [4]. Figure 4 summarises these findings in a diagnostic approach.

In the future, newer methods such as the stimulated copeptin test (with arginine or hypertonic saline) to differentiate between PP and AVP‐D may come into clinical use, both independently and in conjugation with the WDT [12]. However, studies have highlighted several challenges associated with their implementation into widespread clinical use that has limited their accuracy and application [13]. Since the WDT remains the gold standard for diagnosis, this study aimed to improve the currently used cut‐off values; a comparison with copeptin‐based tests was outside the scope of this study.

### 4.1 Limitations

The study was limited by its retrospective design and the risk of clinician selection bias. Although the study analysed 154 indirect water deprivation tests conducted over 9 years, it only included four patients with AVP‐R or partial AVP‐R. Moreover, while 95% of patients were correctly identified without the need for clinician assessment, the cut‐off values still produced equivocal results (*n* = 6) where further testing or clinician insight was required. The findings are also only applicable within the confines of the WDT, further validation is required to establish the diagnostic accuracy of baseline measurements in a less controlled outpatient setting and prospectively in a larger patient population.

## 5 Conclusion

The water deprivation test is the current gold‐standard to diagnose AVP‐D and AVP‐R. This study sought to improve the diagnostic accuracy of the test and demonstrated that a serum sodium cut‐off in tandem with urine osmolality, may yield an improved diagnostic accuracy compared to using the current serum osmolality cut‐off. We recommend that serum sodium be measured in addition to serum osmolality in all indirect water deprivation tests, owing to its reduced variability.

## Conflicts of Interest

The authors declare no conflicts of interest.

## Supporting information

Supporting information.

## Data Availability

The authors have nothing to report.
