# Supplementary material for: Redefining Diagnostic Cut‐Offs for the Indirect Water Deprivation Test
Source: Clin Endocrinol (Oxf). 2024 Dec 5;102(2):149–55. doi: 10.1111/cen.15172 (PMC11694560; doi:10.1111/cen.15172)

***Supplementary Figure****: ROC analyses and Sensitivity/Specificity Curves for Phase 2 Urine Osmolalities: ROC and Sensitivity/Specificity Curves for Urine Osmolality Change in all patients and ROC and Sensitivity/Specificity Curves for Urine Osmolality Change in patients classified as positive (with a diagnosis of AVP-D considered as a positive). Area under the curve (AUC) values have been labelled on the graphs.*


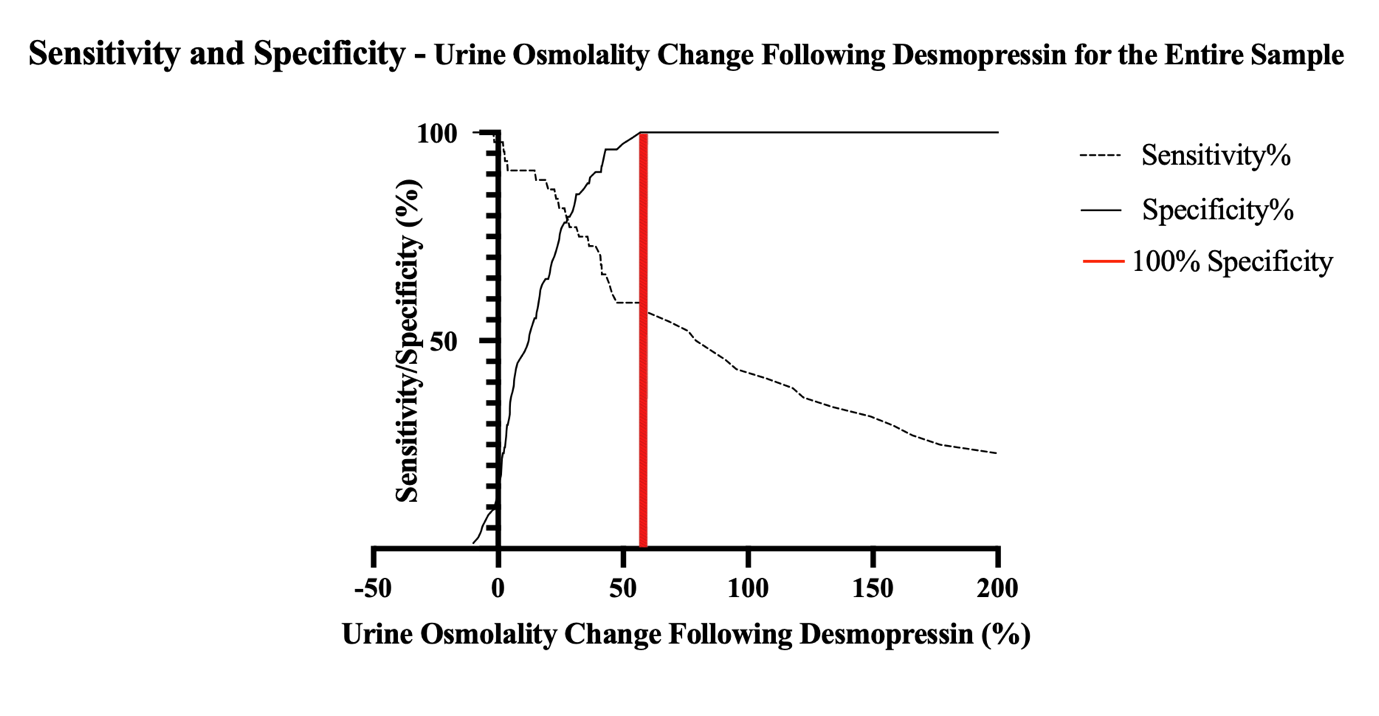

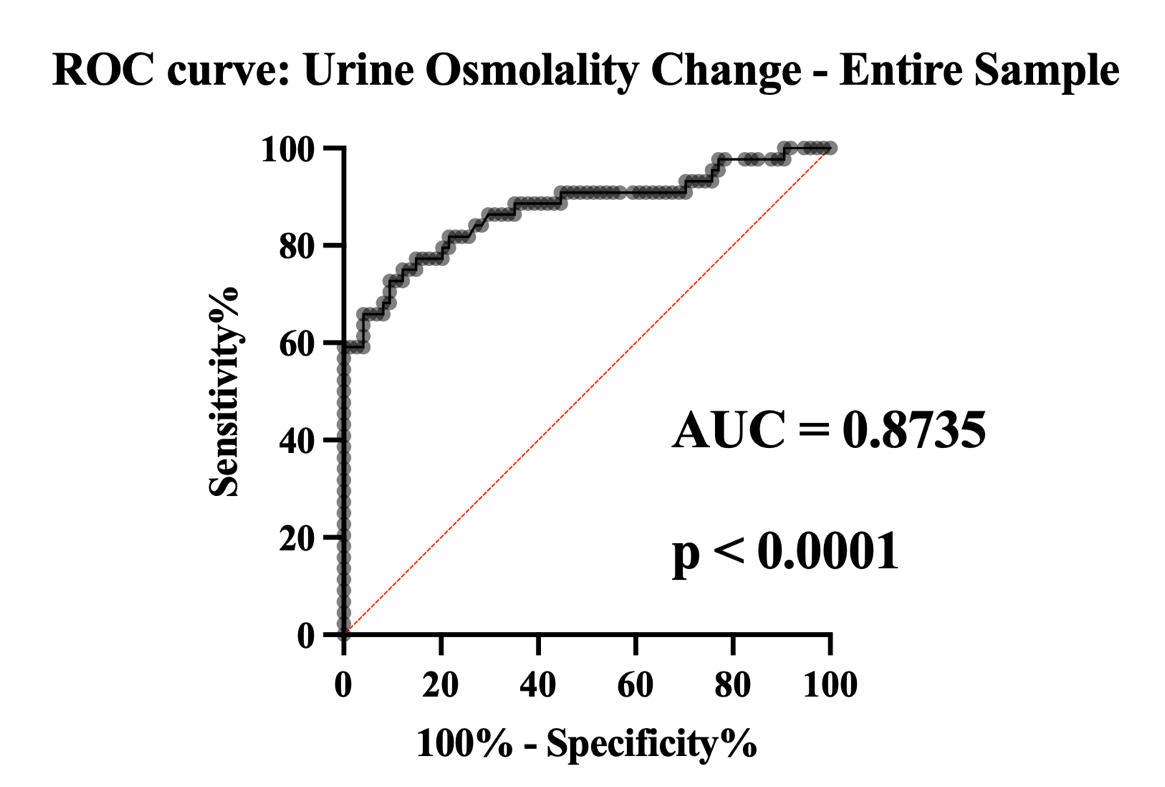

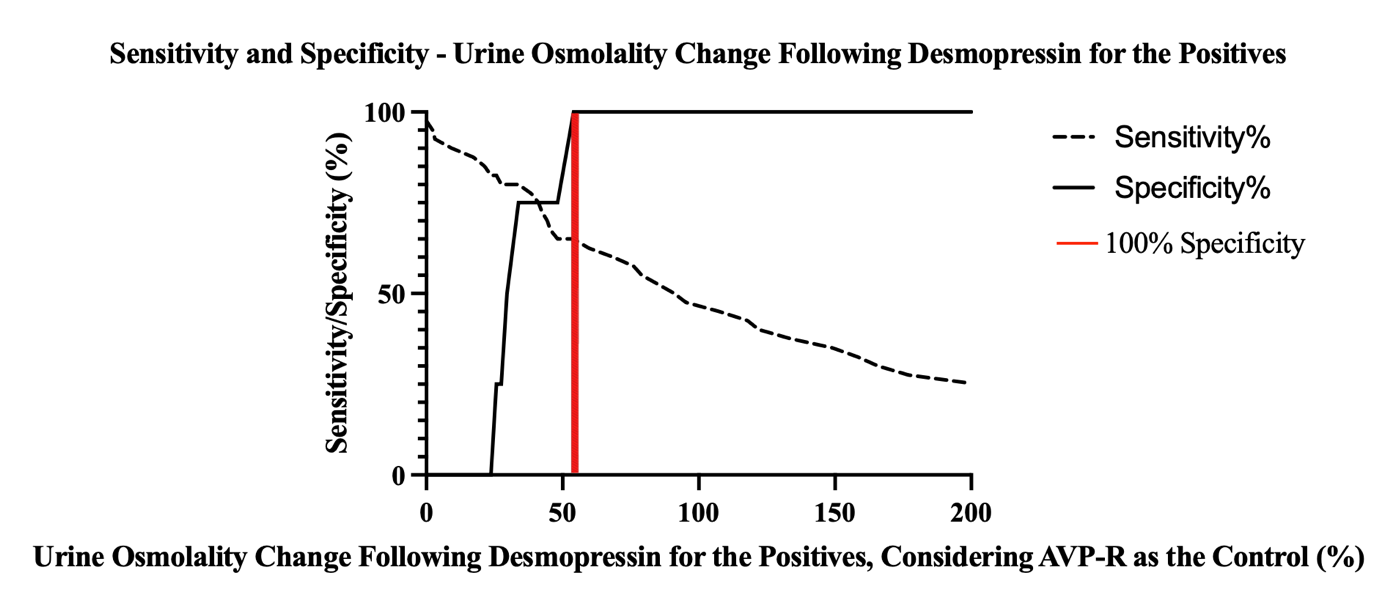

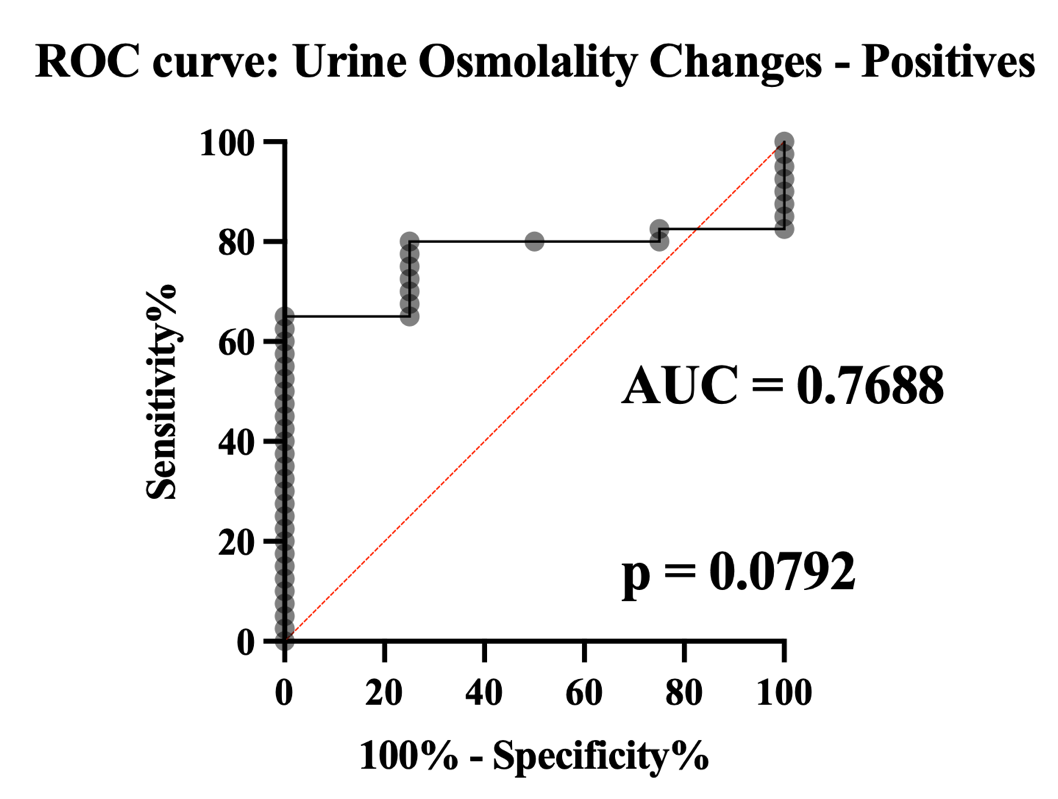

Supplement: Supplementary file 1 — Supporting information. [file CEN-102-149-s001.docx]
